# CovAID: Identification of factors associated with severe COVID-19 in patients with inflammatory rheumatism or autoimmune diseases

**DOI:** 10.3389/fmed.2023.1152587

**Published:** 2023-03-22

**Authors:** Kevin Chevalier, Michaël Genin, Thomas Petit Jean, Jerôme Avouac, Rene-Marc Flipo, Sophie Georgin-Lavialle, Soumaya El Mahou, Edouard Pertuiset, Thao Pham, Amelie Servettaz, Hubert Marotte, Fanny Domont, Pascal Chazerain, Mathilde Devaux, Arsene Mekinian, Jérémie Sellam, Bruno Fautrel, Diane Rouzaud, Esther Ebstein, Nathalie Costedoat-Chalumeau, Christophe Richez, Eric Hachulla, Xavier Mariette, Raphaèle Seror

**Affiliations:** ^1^Department of Rheumatology, Université Paris-Saclay, INSERM UMR 1184: Center for Immunology of Viral Infections and Autoimmune Diseases, Assistance Publique-Hôpitaux de Paris, Hôpital Bicêtre, Le Kremlin-Bicêtre, France; ^2^University of Lille, CHU Lille, ULR 2694–METRICS: Evaluation des Technologies de Santé et des Pratiques Médicales, Lille, France; ^3^AP-HP, Département Innovation et Données, Paris, France; ^4^Cochin Hospital, Rheumatology A, Paris, France; ^5^Université de Lille, Rheumatology, Lille, France; ^6^Tenon Hospital, Internal Medicine, Paris, France; ^7^Hospital Center De Tourcoing, Rheumatology, Tourcoing, France; ^8^Hospital Rene Dubos, Rheumatology, Pontoise, France; ^9^Hospital Sainte Marguerite, Rheumatology, Marseille, France; ^10^Hospital Robert Debré, Internal Medicine, Infectious Diseases and Clinical Immunology, Reims, France; ^11^University Hospital of Saint-Étienne, Rheumatology, Saint-Priest-en-Jarez, France; ^12^University Hospitals Pitié Salpêtrière - Charles Foix, Internal Medicine and Clinical Immunology, Paris, France; ^13^Hopital de la Croix Saint-Simon, Rheumatology and Internal Medicine, Paris, France; ^14^Saint-Germain-en-Laye Intercommunal Hospital Center, Internal Medicine, Poissy, France; ^15^Hospital Saint-Antoine AP-HP, Internal Medicine, Paris, France; ^16^Hospital Saint-Antoine AP-HP, Rheumatology, Paris, France; ^17^Sorbonne Universite – APHP, Pitie Salpetriere Hospital, Department of Rheumatology, Pierre Louis Institute of Epidemiology and Public Health, INSERM UMRS 1136, Paris, France; ^18^Bichat-Claude Bernard Hospital, Internal Medicine, Paris, France; ^19^Bichat-Claude Bernard Hospital, Rheumatology, Paris, France; ^20^Cochin Hospital, Internal Medicine, Paris, France; ^21^Chu Bordeaux - Site Pellegrin, Rheumatology, Bordeaux, France; ^22^Department of Internal Medicine and Clinical Immunology, Referral Centre for Centre for Rare Systemic Autoimmune Diseases North and North-West of France (CeRAINO), CHU Lille, University of Lille, INSERM, U1286 - INFINITE - Institute for Translational Research in Inflammation, Lille, France

**Keywords:** COVID-19, auto-immune diseases, inflammatory rheumatic diseases, rituximab, lupus, vasculitis, rheumatic and musculoskeletal diseases

## Abstract

**Introduction:**

Autoimmune/inflammatory rheumatic diseases (AIRDs) patients might be at-risk of severe COVID-19. However, whether this is linked to the disease or to its treatment is difficult to determine. This study aimed to identify factors associated with occurrence of severe COVID-19 in AIRD patients and to evaluate whether having an AIRD was associated with increased risk of severe COVID-19 or death.

**Materials and methods:**

Two databases were analyzed: the EDS (Entrepôt des Données de Santé, Clinical Data Warehouse), including all patients followed in Paris university hospitals and the French multi-center COVID-19 cohort [French rheumatic and musculoskeletal diseases (RMD)]. First, in a combined analysis we compared patients with severe and non-severe COVID-19 to identify factors associated with severity. Then, we performed a propensity matched score case–control study within the EDS database to compare AIRD cases and non-AIRD controls.

**Results:**

Among 1,213 patients, 195 (16.1%) experienced severe COVID-19. In multivariate analysis, older age, interstitial lung disease (ILD), arterial hypertension, obesity, sarcoidosis, vasculitis, auto-inflammatory diseases, and treatment with corticosteroids or rituximab were associated with increased risk of severe COVID-19. Among 35,741 COVID-19 patients in EDS, 316 having AIRDs were compared to 1,264 Propensity score-matched controls. AIRD patients had a higher risk of severe COVID-19 [aOR = 1.43 (1.08–1.87), p = 0.01] but analysis restricted to rheumatoid arthritis and spondyloarthritis found no increased risk of severe COVID-19 [aOR = 1.11 (0.68–1.81)].

**Conclusion:**

In this multicenter study, we confirmed that AIRD patients treated with rituximab or corticosteroids and/or having vasculitis, auto-inflammatory disease, and sarcoidosis had increased risk of severe COVID-19. Also, AIRD patients had, overall, an increased risk of severe COVID-19 compares general population.

## Introduction

The COVID-19 pandemic has put the medical world in a new situation and has changed how we treat patients. In this context, access to the health database from all university hospitals of the Paris region (APHP, Assistance Publique–Hôpitaux de Paris), named Entrepôt des Données de Santé (EDS, Clinical Data Warehouse), has been set up for COVID-19-related research projects (EDS-COVID). This database captures all hospitalized cases of COVID-19 in these 39 university hospitals and allows for different kinds of studies in several populations.

One of risk population identified during the pandemic was patients with autoimmune/inflammatory rheumatic diseases (AIRDs). However, whether this increased risk was linked to the disease or with specific treatment was difficult to determine, and only anti-CD20 therapeutics, such as rituximab, have been associated with extensive risk of more severe COVID-19 disease (1). Moreover, despite the unprecedented speed of vaccine development against COVID-19, the emergence of new variants is very probable, with a “sword of Damocles” hanging over us (2). These variants threaten to overturn the significant progress made so far increasing resistance to vaccines or monoclonal antibody therapeutics (3, 4) which is very important in patients whom vaccines are not effective because of immunosuppressive treatments (such as anti-CD20 therapies).

Because most cases are identified by diagnostic testing and hospitalization, estimates of the incidence of this disease are greatly underestimated and biased toward moderate to severe disease forms. Mild forms treated in outpatient clinics, or even not treated, were rarely identified by health databases. For AIRDs, in France, a national cohort was set up at the beginning of the epidemic [French rheumatic and musculoskeletal diseases (RMD) COVID-19 cohort] (5). This cohort has collected more than 1,200 cases of COVID in AIRD patients. In this cohort, approximately 50% of cases are the benign forms, which are difficult to capture from other sources.

The accessibility of theses two databases for scientific projects is a unique opportunity to provide answers to these questions. The objectives of this study were to identify the factors associated with the occurrence of severe or moderate-to-severe COVID-19 in patients with AIRDs, by using a combination of these 2 databases, and to evaluate whether having an AIRD is associated with an increased risk of severe COVID-19, in a case–control study within the EDS.

## Materials and methods

### Methodology overview

This study had two parts:

1. The first part aimed to identify factors associated with COVID-19 severity. For that purpose, we performed a comparative observational study of patients with COVID-19 and an AIRD by combining data from the EDS-COVID and RMD cohorts.
2. The second part aimed to compare COVID-19 severity (risk of death) in patients with an AIRD and controls not having these underlying diseases. For that purpose, we performed a case–control study within the EDS-COVID database.

### Data source

The data were provided by two registers: the EDS-COVID database based on the EDS (6) and the French RMD COVID-19 cohort (5). Both cohorts are described in Supplementary material.

### Ethics

This present study was approved by the institutional review board (APHP Scientific and Ethical Committee, authorization no. CSE 20-60_CovAID) from the Scientific and Ethical Committee of the AP-HP and by the CNIL. All participants included were informed about the use of their data for research. Patients who expressed an objection to the use of their data were excluded from this study.

### Patients

For the EDS-COVID, we performed a systematic electronic search based on International Classification of Disease, 10th revision (ICD-10) codes to identify patients with an AIRD of interest (list provide in Supplementary Table 1). Patients were included if they had confirmed COVID-19 [i.e., positive COVID-19 PCR or serology result or a chest CT-scan interpreted as possible or certain COVID-19 (7)] and a confirmed diagnosis of one of the AIRDs of interest. Patients were excluded if no information was found about the COVID-19 status (i.e., no proof of infection) or any underlying immune disease. All medical files were reviewed by one of the authors (KC) to validate the diagnosis. Data were retrospectively analyzed.

For the French RMD COVID-19 cohort, patients were enrolled if they had one of the AIRDs of interest and a highly suspected or confirmed diagnosis of COVID-19 (i.e., positive PCR or serology result or a chest CT-scan interpreted as possible or certain COVID-19, or anosmia or sudden ageusia in the absence of rhinitis or nasal obstruction, or typical clinical signs of COVID-19: cough, fever, nose/throat symptoms, digestive symptoms without any other diagnosis, flu-like syndrome in a patient with recent close contact with a known COVID-19-positive patient) (5).

For both cohorts, patients were included if COVID-19 was diagnosed between the start of the pandemic until 1 September 2020. We excluded patients under 18 years old. AIRDs were classified into groups of diseases (details in Supplementary Table 1).

### Outcomes

Endpoints rely on COVID-19 severity according to WHO criteria (Supplementary Table 2). COVID-19 was defined as ambulatory mild disease if the WHO score was 1–3, moderate if the score was 4 or 5, and severe if the WHO score was 6–10 [hospitalization in an intensive care unit (ICU) and/or death]. We considered death from any cause within 90 days after COVID-19 diagnosis.

#### Statistical analyses

##### Identification of factors associated with COVID-19 severity among RMD patients: Combined analysis of EDS-COVID and RMD cohorts

To eliminate duplicates, data from EDS-COVID and RMD COVID-19 cohorts were pooled and then cross-referenced according to the birthdate, sex, date of first symptoms, date of diagnosis, date of hospitalization, and underlying disease. For this part, all medical files were reviewed to collect and confirm the following data: demographics data, comorbidities [interstitial pneumonia, chronic obstructive pulmonary disease (COPD), cardiovascular disease, stroke, diabetes, obesity, hypertension, smoking status, and cancer], underlying disease and its activity, AIRD treatments, dates of first symptoms, evolution, and outcome of COVID-19 (hospitalization, admission to an ICU, date of hospital discharge, and death; Supplementary Table 2).

For this part, the primary outcome was defined as severe COVID-19. Secondary outcomes were moderate-to-severe COVID-19. For these analyses, patients with severe (or moderate-to-severe) COVID-19 were compared to those with non-severe (or ambulatory mild) disease. Also, among patients with moderate-to-severe disease, patients with severe COVID-19 were compared to those with moderate disease to identify risk factors associated with ICU admission and/or death. Categorical variables are expressed as number (percentage) and quantitative variables as mean (SD). Comparisons of patients with severe versus non-severe disease and survivors versus non-survivors were assessed with logistic regression models. In case of cell frequency<5, a penalized logistic regression (Firth’s method) was used. Odds ratios (ORs) and their 95% confidence intervals (CIs) were calculated as the effect size. Factors associated with severity and death status on univariate analyses (*p* < 0.05) were introduced into multivariable penalized logistic regression models with a forward stepwise selection procedure (entrance criterion=0.05) to limit overfitting.

##### COVID-19 severity in AIRD patients compared to the general population: A propensity score-matched case–control study with the EDS-COVID database

For the second part of the study, we performed a case–control study within the EDS-COVID database. Cases were AIRD patients identified and manually confirmed in the EDS-COVID database. Non-RMD controls were identified in the EDS-COVID database, after excluding all patients with an ICD-10 code for an AIRD (Supplementary Table 1).

Because the EDS-COVID cohort included>35,000 patients, COVID-19 and comorbidity diagnoses could not be reviewed manually for all patients; comorbidity diagnoses were based on structured (ICD-10 codes) and/or unstructured data (rule-based phenotyping on clinical notes). Regarding the structured data approach, a set of ICD-10 codes was used for each comorbidity (Supplementary Table 3) and allowed for tagging each patient for this specific comorbidity. To extract the comorbidity status from clinical notes, the PyMedExt Python library (8) was used to extract both comorbidities and associated modifiers, namely, negation, family history context and hypothesis (9). The library leverages a set of regular expression to extract comorbidity mentions, along with a rule-based algorithm to qualify each mention. We considered only positive and certain occurrence concerning the patient. Finally, we considered that a given patient had a given comorbidity if we found at least one associated ICD-10 code or at least 2 occurrences of the comorbidity in the patient’s clinical notes. For each patient, we considered only ICD-10 codes and clinical notes edited before the COVID-19 diagnosis. For the RMD case cohort, those comorbidities were also extracted manually by KC who had access to each patient’s records. Thus, for this cohort, performance of this method (sensitivity, specificity, precision and F1 score; definitions available in Supplementary material 2 and Supplementary Table 4) were assessed by using manual identification as a reference (Supplementary Figure 1).

For cases and controls, the COVID-19 diagnosis was based on one of the following: positive PCR or serology result, chest-imaging report confirming a diagnosis of COVID-19, or COVID-19 ICD-10 code: U071 (excluding U0712 and U0713 which is the code for “SARS-CoV-2 carrier, asymptomatic” and “Other examinations and observations related to the outbreak COVID-19,” respectively). We then extracted dates of COVID-19 diagnosis. If multiple dates were available, the first was used.

Autoimmune/inflammatory rheumatic diseases patients (cases) and non-AIRD patients (controls) were matched on a propensity score. The propensity score included age, sex, and comorbidities and was estimated by means of multivariable logistic regression. These comorbidities were chosen because they were identified as COVID-19 severity risk factors at the time of the analysis (5, 10, 11). The two groups were matched (1:4) by using an optimal algorithm with caliper width 0.2 SD of logit for the propensity score (12, 13). To assess the bias reduction, absolute standardized differences (ASDs) were computed before and after matching. An ASD > 10% was considered a significant difference (14).

For this case–control study, the primary outcome was severe COVID-19 and secondary outcomes were ICU admission and death from any cause within 90 days.

ORs for severe COVID-19, death, and ICU admission were estimated by conditional logistic regression. If a confounding factor still had an ASD > 10% after matching, it was introduced in this previous model as an adjustment factor (15).

### Sensitivity analyses

Because each AIRD might not have the same risk of severity or death, we performed a separate analysis of only patients with chronic inflammatory arthritis (CIA) including spondyloarthritis (SA), psoriasis arthritis (PsA) and rheumatoid arthritis (RA) to analyze whether these diseases were associated with increased risk of COVID-19 severity. Thus, we used the same analysis (identification of factors associated with COVID-19 severity and comparison with the general population) for the CIA population alone.

## Results

### Identification of factors associated with COVID-19 severity among RMD patients: Combined analysis of EDS-COVID and RMD cohorts

#### Patients

**FIGURE 1:**
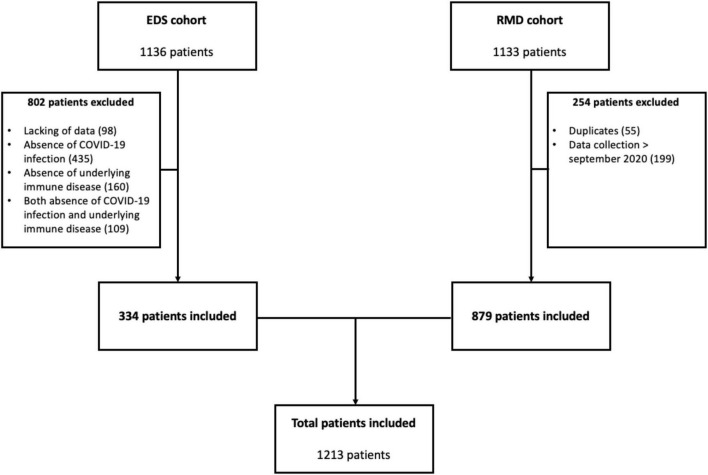
Flow chart of patient inclusion. EDS, Entrepôt des Données de Santé; RMD, rheumatic and musculoskeletal diseases.

By 1 September 2020, 1,136 potential AIRD COVID-19 patients were identified within the EDS database, and after medical chart review 334 patients were included in the study (Figure 1). Also, 1,133 adults were identified from the French RMD COVID-19 cohort and among them 879 patients were included in the final analysis. Thus, 1,213 patients were included in this part of the study (Table 1). Eight hundred and twelve patients (66.9%) were diagnosed thanks to PCR or serology and 95 (0.8%) were diagnosed thanks to the typical CT scan pattern. The other patients were diagnosed thanks to a typical clinical symptomatology (i.e., fever, flue-like symptoms, anosmia, ageusia).

**TABLE 1:** Characteristics of RMD patients in the pooled analysis of EDS and RMD COVID-19 French cohorts (*N* = 1213).

Patient characteristics	RMD patients in EDS and RMD COVID-19 cohorts *N* = 1213
Age	58.2 (17.2)
Sex: male	412 (34.0%)
**Comorbidities**	
Interstitial lung disease	58 (4.78%)
COPD	58 (4.78%)
Asthma	86 (7.09%)
Coronary heart diseases	172 (14.2%)
Stroke	56 (4.62%)
Diabetes	143 (11.8%)
Obesity	181 (19.2%)
Hypertension	382 (31.5%)
Smoking	106 (8.74%)
Cancer	81 (6.68%)
Number of patients with at least 1 comorbidity	726 (65.1%)
**Underlying disease**	
Chronic inflammatory arthritis	690 (56.9%)
Auto-inflammatory disease	34 (2.81%)
Connective tissue diseases	224 (18.5%)
Sarcoidosis	64 (5.28%)
Vasculitis	167 (13.8%)
Other	33 (2.72%)
**Ongoing rheumatic diseases or AID treatments**	
Corticosteroids	435 (35.9%)
Dose	6.00 [5.00;10.0]
NSAIDs	102 (8.41%)
Colchicine	36 (2.97%)
Hydroxychloroquine	116 (9.56%)
Methotrexate	397 (32.7%)
Leflunomide	46 (3.79%)
Salazopyrine	13 (1.07%)
Mycophenolate mofetil/mycophenolic acid	35 (2.89%)
Azathioprine	20 (1.65%)
IVIg	7 (0.58%)
Biologics	474 (39.1%)
Anti-TNF alpha	281 (23.4%)
Anti IL-6	33 (2.75%)
Anti IL-1	9 (0.75%)
Anti-IL-17	36 (3.00%)
Abatacept	22 (1.83%)
Rituximab	59 (4.92%)
JAK inhibitor	31 (2.56%)
Other	20 (1.67%)

COPD, chronic obstructive pulmonary disease; IVIg, intravenous immunoglobulins; JAK, Janus kinase; NSAIDs, non-steroidal anti-inflammatory drugs; TNF, tumor necrosis factor; IL, interleukin.

The mean (SD) age of patients was 58.2 (17.2) years and many were women (*n* = 800, 66.0%). The most frequent underlying diseases were CIA (56.9%), connective tissue disease (56.9%) and vasculitis (13.8%). At least one comorbidity was recorded in 726 (65.1%) patients. Among the 1,213 patients, 195 (16.1%), 595 (49.1%), and 422 (34.8%) experienced severe, moderate and mild COVID-19, respectively (Table 2).

**TABLE 2:** Factors associated with odds of severe COVID-19 in autoimmune/inflammatory rheumatic disease (AIRD) patients: Pooled analysis of EDS-COVID and the French RMD cohort patients.

	Overall	Patients with mild or moderate COVID-19	Patients with severe COVID-19	Univariate analysis		*N*	Multivariate analysis	
	***N*** **= 1213**	***N*** **= 1018**	***N*** **= 195**	**OR (95% CI)**	***p*** **-Value**		**aOR 95% CI**	***p*** **-Value**
**Patient characteristics**								
Age	58.2 (17.2)	55.9 (16.7)	70.3 (14.3)	1.06 [1.05–1.07]	<0.001	1,213	**1.05 [1.03–1.07]**	**<0.001**
Sex: male	412 (34.0%)	323 (31.7%)	89 (45.9%)	1.82 [1.33–2.49]	<0.001	1,212	**1.69 [1.08–2.64]**	**0.0206**
**Comorbidities**								
Interstitial lung disease	58 (4.78%)	38 (3.73%)	20 (10.3%)	2.97 [1.67–5.15]	<0.001	1,213	**2.94 [1.34–6.34]**	**0.0077**
COPD	58 (4.78%)	38 (3.73%)	20 (10.3%)	2.97 [1.67–5.15]	<0.001	1,213		
Asthma	86 (7.09%)	68 (6.68%)	18 (9.23%)	1.45 [0.82–2.43]	0.1922	1,213		
Coronary heart disease	172 (14.2%)	114 (11.2%)	58 (29.7%)	3.36 [2.33–4.82]	<0.001	1,213		
Stroke	56 (4.62%)	38 (3.73%)	18 (9.23%)	2.65 [1.46–4.67]	0.0018	1,213		
Diabetes	143 (11.8%)	96 (9.43%)	47 (24.1%)	3.06 [2.06–4.49]	<0.001	1,213		
Obesity	181 (19.2%)	143 (17.8%)	38 (27.7%)	1.79 [1.17–2.68]	0.0076	942	**2.09 [1.26–3.43]**	**0.0044**
Hypertension	382 (31.5%)	268 (26.3%)	114 (58.5%)	3.93 [2.87–5.40]	<0.001	1,213	**1.81 [1.13–2.89]**	**0.0128**
Smoking	106 (8.74%)	90 (8.84%)	16 (8.21%)	0.94 [0.53–1.59]	0.8332	1,213		
Cancer	81 (6.68%)	60 (5.89%)	21 (10.8%)	1.95 [1.14–3.23]	0.0157	1,213		
No. of patients with at least 1 comorbidity	726 (65.1%)	555 (59.9%)	171 (90.5%)	6.20 [3.87–10.52]	<0.001	1,115		
Underlying disease					<0.001	1212		
Chronic inflammatory arthritis	690 (56.9%)	618 (60.8%)	72 (36.9%)	Ref.			Ref.	
Auto-inflammatory disease	34 (2.81%)	29 (2.85%)	5 (2.56%)	1.59 [0.56–3.80]	0.3571		**3.91 [1.2–11.32]**	**0.0251**
Connective tissue diseases	224 (18.5%)	190 (18.7%)	34 (17.4%)	1.54 [0.99–2.37]	0.0558		**1.13 [0.62–2.01]**	**0.6919**
Sarcoidosis	64 (5.28%)	40 (3.93%)	24 (12.3%)	5.16 [2.93–8.97]	<0.001		**5.19 [2.15–12.3]**	**0.0003**
Vasculitis	167 (13.8%)	111 (10.9%)	56 (28.7%)	4.32 [2.89–6.46]	<0.001		**1.8 [1.02–3.16]**	**0.0435**
Other	33 (2.72%)	29 (2.85%)	4 (2.05%)	1.30 [0.41–3.29]	0.6234		**0.35 [0.06–1.41]**	**0.1503**
**Ongoing rheumatic diseases or AIRD treatments**								
Corticosteroids	435 (35.9%)	318 (31.2%)	117 (60.0%)	3.29 [2.41–4.52]	<0.001	1,213	**2.47 [1.58–3.87]**	**0.0001**
Dose	6.0 [5.0–10.0]	5.0 [5.0–10.]	7.3 [5.0–13.1]	1.01 [1.00–1.03]	0.0229	431		
NSAIDs	102 (8.41%)	98 (9.63%)	4 (2.05%)	0.22 [0.07–0.51]	<0.001	1,213		
Colchicine	36 (2.97%)	31 (3.05%)	5 (2.56%)	0.91 [0.32–2.10]	0.8298	1,213		
Hydroxychloroquine	116 (9.56%)	101 (9.92%)	15 (7.69%)	0.78 [0.43–1.32]	0.3612	1,213		
Methotrexate	397 (32.7%)	349 (34.3%)	48 (24.6%)	0.63 [0.44–0.89]	0.0077	1,213		
Leflunomide	46 (3.79%)	44 (4.32%)	2 (1.03%)	0.28 [0.06–0.84]	0.0201	1,213	**0.13 [0–0.97]**	**0.0453**
Salazopyrine	13 (1.07%)	11 (1.08%)	2 (1.03%)	1.13 [0.22–3.87]	0.8618	1,213		
Mycophenolate Mofetil/mycophenolic acid	35 (2.89%)	31 (3.05%)	4 (2.05%)	0.74 [0.23–1.82]	0.5345	1,213		
Azathioprine	20 (1.65%)	16 (1.57%)	4 (2.05%)	1.43 [0.44–3.79]	0.5219	1,213		
IVIg	7 (0.58%)	5 (0.49%)	2 (1.03%)	2.38 [0.43–9.95]	0.2881	1,213		
Biologics	474 (39.1%)	430 (42.2%)	44 (22.6%)	0.40 [0.28–0.57]	<0.001	1,213		
Anti-TNF alpha	281 (23.4%)	271 (26.9%)	10 (5.21%)	0.16 [0.08–0.28]	<0.001	1,199		
Anti IL-6	33 (2.75%)	30 (2.98%)	3 (1.56%)	0.59 [0.16–1.59]	0.3256	1,199		
Anti IL-1	9 (0.75%)	7 (0.70%)	2 (1.04%)	1.75 [0.33–6.61]	0.4678	1,199		
Anti-IL17	36 (3.00%)	34 (3.38%)	2 (1.04%)	0.37 [0.08–1.12]	0.0831	1,199		
Abatacept	22 (1.83%)	21 (2.09%)	1 (0.52%)	0.36 [0.04–1.41]	0.1617	1,199		
Rituximab	59 (4.92%)	37 (3.67%)	22 (11.5%)	3.42 [1.95–5.86]	<0.001	1,199	**4.05 [1.96–8.27]**	**0.0002**
JAK inhibitor	31 (2.56%)	25 (2.46%)	6 (3.08%)	1.34 [0.51–3.03]	0.5275	1,213		
Other	20 (1.67%)	19 (1.89%)	1 (0.52%)	0.40 [0.04–1.57]	0.2159	1,199		

COPD, chronic obstructive pulmonary disease; IVIg, intravenous immunoglobulins; JAK, Janus kinase; NSAIDs, non-steroidal anti-inflammatory drugs; OR, odds ratio-aOR: adjusted odds ratio; 95% CI, 95% confidence interval. Bold values represent the significantive value.

#### Factors associated with COVID-19 severity

On univariate analyses (Table 2), the probability of severe versus mild or moderate COVID-19 was associated with mean age [70.3 (14.3) vs. 55.9 (16.7) years, *p* < 0.001], male sex (45.9% vs. 31.7%, *p* < 0.001), at least one comorbidity, history of interstitial lung disease (ILD), COPD, coronary heart disease. stroke, diabetes, obesity, arterial hypertension, and cancer. Regarding underlying diseases, sarcoidosis and vasculitis were associated with severe COVID-19. The use of corticosteroids (yes vs. no or ≥10 mg vs. <10 mg) was associated with poor outcomes, as was rituximab. All variables were entered in a multivariate model, for corticosteroids to avoid collinearity only corticosteroids use (but not the dose) was retained. On multivariate analysis, severe COVID-19 remained associated with age [adjusted OR (aOR) = 1.05 (95% CI 1.03–1.07), for each additional year], male sex [aOR = 1.69 (1.08–2.64)], history of ILD [aOR = 2.94 (1.34–6.34)], arterial hypertension [aOR = 1.81 (1.13–2.89)], obesity [aOR = 2.09 (1.26–3.43)], sarcoidosis [aOR = 5.19 (2.15–12.3)], vasculitis [aOR = 1.8 (1.02–3.16)], auto-inflammatory disease [OR = 3.91 (1.2–11.32)], corticosteroids treatment [aOR = 2.47 (1.58–3.87)] and rituximab treatment [aOR = 4.05 (1.96–8.27)]. By contrast, treatment with leflunomide was associated with better outcome [aOR = 0.13 (0–0.97)].

In patients with CIA (Supplementary Table 5), multivariate analyses revealed the same factors as those associated with severe COVID-19: older age [aOR = 1.05 (95% CI 1.03–1.07)], male sex [aOR = 2.19 (1.25–3.87)], history of ILD [aOR = 4.26 (1.22–14.39)] or arterial hypertension [aOR = 2.68 (1.48–4.94)], and use of corticosteroids [aOR = 2.44 (1.38–4.30)] or rituximab [aOR = 5.20 (1.83–14.01)].

#### Factors associated with death

Among the 790 patients admitted for moderate to severe COVID-19, survival status at 90 days was available for 590 (Table 3): 116 (19.7%) died within the 90 days after COVID-19 diagnosis. On univariate analyses, among hospitalized patients, probability of death was associated with older age; a history of COPD, coronary heart disease or arterial hypertension; a diagnosis of vasculitis; and corticosteroids or rituximab treatment. However, probability of survival was also associated with methotrexate or anti-tumor necrosis factor α treatment. On multivariable analysis, probability of death remained associated with older age [aOR = 1.06 (1.04–1.08)], a history of ILD [aOR = 2.34 (1.10–4.84)] or arterial hypertension [aOR = 1.64 (1.00–2.70)] and use of corticosteroids [aOR = 1.67 (1.05–2.66)] and rituximab [aOR = 3.32 (1.45–7.49)]. Also, methotrexate treatment remained significantly associated with better outcomes [aOR of death = 0.43 (0.25–0.73)]. Analyses restricted to CIA led to the identification of the same risk factors, except for methotrexate, which was longer significantly associated with better outcomes (Supplementary Table 6).

**TABLE 3:** Factors associated with odds of death from COVID-19 in AIRD patients: Pooled analysis of EDS-COVID and French RMD cohorts.

				Univariate analyses			Multivariate analyses	
	**Overall**	**Survivor**	**Non-survivor**	**OR (95% CI)**	***p*** **-Value**	***N***	**aOR 95% CI**	***p*** **-Value**
	***N*** **= 590**	***N*** **= 474**	***N*** **= 116**					
**Patients characteristics**								
Age	66.3 (16.0)	63.9 (16.0)	76.3 (11.9)	1.06 [1.04–1.08]	<0.001	590	**1.06 [1.04–1.08]**	<**0.001**
Sex: male	221 (37.5%)	173 (36.5%)	48 (41.7%)	1.25 [0.82–1.88]	0.2945	589		
**Comorbidities**								
Interstitial lung disease	46 (7.80%)	32 (6.75%)	14 (12.1%)	1.93 [0.97–3.65]	0.059	590	**2.34 [1.10–4.84]**	**0.0283**
COPD	43 (7.29%)	28 (5.91%)	15 (12.9%)	2.39 [1.22–4.55]	0.0124	590		
Asthma	44 (7.46%)	35 (7.38%)	9 (7.76%)	1.09 [0.49–2.22]	0.8147	590		
Coronary heart disease	151 (25.6%)	107 (22.6%)	44 (37.9%)	2.10 [1.36–3.22]	<0.001	590		
Stroke	48 (8.14%)	34 (7.17%)	14 (12.1%)	1.81 [0.92–3.40]	0.0852	590		
Diabetes	120 (20.3%)	89 (18.8%)	31 (26.7%)	1.59 [0.98–2.52]	0.0584	590		
Obesity	100 (24.5%)	76 (23.2%)	24 (29.6%)	1.40 [0.81–2.38]	0.2253	408		
Hypertension	284 (48.1%)	206 (43.5%)	78 (67.2%)	2.65 [1.74–4.09]	<0.001	590	**1.64[1.00–2.70]**	**0.048**
Smoking	40 (6.78%)	32 (6.75%)	8 (6.90%)	1.07 [0.46–2.24]	0.8725	590		
Cancer	65 (11.0%)	49 (10.3%)	16 (13.8%)	1.41 [0.76–2.52]	0.2695	590		
No. of patients with at least 1 comorbidity	461 (83.5%)	354 (80.6%)	107 (94.7%)	3.99 [1.88–10.06]	<0.001	552		
**Underlying disease**					0.008	590		
Chronic inflammatory arthritis	253 (42.9%)	216 (45.6%)	37 (31.9%)	Ref.				
Auto-inflammatory disease	15 (2.54%)	11 (2.32%)	4 (3.45%)	2.26 [0.65–6.72]	0.1863			
Connective tissue diseases	128 (21.7%)	104 (21.9%)	24 (20.7%)	1.35 [0.77–2.36]	0.2932			
Sarcoidosis	49 (8.31%)	38 (8.02%)	11 (9.48%)	1.72 [0.79–3.55]	0.1638			
Vasculitis	128 (21.7%)	89 (18.8%)	39 (33.6%)	2.55 [1.53–4.26]	<0.001			
Other	17 (2.88%)	16 (3.38%)	1 (0.86%)	0.52 [0.06–2.20]	0.4219			
**Ongoing rheumatic diseases or AIRD treatments**								
Corticosteroids	295 (50.0%)	222 (46.8%)	73 (62.9%)	1.92 [1.27–2.92]	0.0019	590	**1.67 [1.05–2.66]**	**0.0302**
Dose	7.00 [5.00–10.0]	5.00 [5.00–10.0]	7.50 [5.00–15.0]	1.02 [1.00–1.03]	0.043	291		
NSAIDs	23 (3.90%)	22 (4.64%)	1 (0.86%)	0.26 [0.03–1.03]	0.0555	590		
Colchicine	21 (3.56%)	16 (3.38%)	5 (4.31%)	1.37 [0.46–3.48]	0.5414	590		
Hydroxychloroquine	55 (9.32%)	48 (10.1%)	7 (6.03%)	0.60 [0.25–1.26]	0.1893	590		
Methotrexate	178 (30.2%)	158 (33.3%)	20 (17.2%)	0.42 [0.25–0.70]	<0.001	590	**0.43 [0.25–0.73]**	**0.0015**
Leflunomide	18 (3.05%)	17 (3.59%)	1 (0.86%)	0.34 [0.04–1.37]	0.1443	590	**0.08 [0.00–0.67]**	**0.0127**
Salazopyrine	6 (1.02%)	5 (1.05%)	1 (0.86%)	NA	NA	590		
Mycophenolate mofetil/mycophenolic acid	20 (3.39%)	17 (3.59%)	3 (2.59%)	0.81 [0.21–2.32]	0.7111	590		
Azathioprine	14 (2.37%)	13 (2.74%)	1 (0.86%)	0.44 [0.05–1.84]	0.2987	590		
IVIg	7 (1.19%)	5 (1.05%)	2 (1.72%)	NA	NA	590		
Biologics	143 (24.2%)	119 (25.1%)	24 (20.7%)	0.79 [0.47–1.27]	0.3347	590		
Anti-TNF alpha	53 (9.14%)	49 (10.5%)	4 (3.51%)	0.34 [0.11–0.83]	0.0155	580		
Anti IL-6	9 (1.55%)	7 (1.50%)	2 (1.75%)	NA	NA	580		
Anti IL-1	6 (1.03%)	4 (0.86%)	2 (1.75%)	NA	NA	580		
Anti-IL17	8 (1.38%)	8 (1.72%)	0 (0.00%)	NA	NA	580		
Abatacept	10 (1.72%)	9 (1.93%)	1 (0.88%)	0.64 [0.07–2.80]	0.5905	580		
Rituximab	40 (6.90%)	27 (5.79%)	13 (11.4%)	2.13 [1.04–4.15]	0.0387	580	**3.32 [1.45–7.49]**	**0.0051**
JAK inhibitor	10 (1.69%)	7 (1.48%)	3 (2.59%)	1.92 [0.46–6.58]	0.34	590		
Other	7 (1.21%)	7 (1.50%)	0 (0.00%)	NA	NA	580		

COPD, chronic obstructive pulmonary disease; IVIg, intravenous immunoglobulins; JAK, Janus kinase; NA, not available; NSAIDs, non-steroidal anti-inflammatory drugs; OR, odds ratio; aOR, adjusted odds ratio; 95% CI, 95% confidence interval; TNF, tumor necrosis factor; IL, interleukin. Bold values represent the significantive value.

### COVID-19 severity in AIRD patients compared to the general population: Propensity score-matched case–control study

Within the EDS-COVID cohort, we identified 35,741 adults with a diagnosis of COVID-19 according to our 4 pre-specified criteria. We identified 316 of the 334 (94.6%) previously described AIRD patients and compared them to 1,264 propensity score-matched controls (Table 4). Before matching, the controls were younger, more often male and had fewer comorbidities then AIRD patients. After the matching, the mean (SD) age was 66.3 (16.8) years for AIRDs patients and 68.7 (18.9) years for controls. The comorbidities were well-balanced between the cases and controls except for ILD (ASD = 0.14), arterial hypertension (ASD = 0.11), and cancer (ASD = 0.15). After conditional logistic regression (adjusted for age at COVID-19, arterial hypertension, ILD, and cancer), severe COVID-19 occurred in 118 (37.3%) AIRD patients and 384 (30.4%) controls [aOR for severe COVID-19 = 1.43 (1.08–1.87), *p* = 0.01] and death in 56 (19.6%) AIRD patients and 221 (17.5%) controls [aOR = 1.33 (0.95–1.86), *p* = 0.095]. Admission to an ICU was also numerically but not significantly more frequent in AIRD patients (*n* = 56, 17.7%) than controls (163, 12.9%) [aOR = 1.36 (0.96–1.93), *p* = 0.085].

**TABLE 4:** Odds of severe COVID-19: Comparison between patients with or without AIRDs: case–control study within EDS database.

	Before PS matching		After PS matching					
	**AIRD patients (*N* = 316)**	**Controls (*N* = 35425)**	**AIRD patients (*N* = 316)**	**Controls (*N* = 1264)**	**ASD**	**aOR**	**95% CI**	***p*** **-Value**
Severe COVID-19 infection	118 (37.3%)	6439 (18.2%)	118 (37.3%)	384 (30.4%)		1.43	[1.08–1.87]	0.01
Death	62 (19.6%)	3235 (9.1%)	62 (19.6%)	221 (17.5%)		1.33	[0.95–1.86]	0.10
ICU admission	56 (17.7%)	3204 (9.0%)	56 (17.7%)	163 (12.9%)		1.36	[0.96–1.93]	0.09
**Patients characteristics**								
Age	66.3 (16.8)	55.4 (21.1)	66.3 (16.8)	68.7 (18.9)	0.26			
Sex: male	121 (38.3%)	16676 (47.1%)	121 (38.3%)	519 (41.1%)	0.03			
**Comorbidities**								
Interstitial lung disease	42 (13.3%)	376 (1.1%)	42 (13.3%)	140 (11.1%)	0.14			
COPD	29 (9.2%)	1543 (4.4%)	29 (9.2%)	119 (9.4%)	0.03			
Cardiovascular diseases	119 (37.7%)	5751 (16.2%)	119 (37.7%)	503 (39.8%)	0.05			
Stroke	53 (16.8%)	2887 (8.1%)	53 (16.8%)	220 (17.4%)	0.07			
Diabetes	74 (23.4%)	5123 (14.5%)	74 (23.4%)	324 (25.6%)	0.03			
Obesity	84 (26.6%)	3730 (10.5%)	84 (26.6%)	349 (27.6%)	0.06			
Hypertension	180 (57.0%)	10069 (28.4%)	180 (57.0%)	767 (60.7%)	0.11			
Smoking	37 (11.7%)	1525 (4.3%)	37 (11.7%)	157 (12.4%)	0.01			
Cancer	66 (20.9%)	3690 (10.4%)	66 (20.9%)	275 (21.8%)	0.15			

PS, propensity score; ASD, absolute standardized difference; COPD, chronic obstructive pulmonary disease, ICU, intensive care unit; aOR, adjusted odds ratio; 95% CI, 95% confidence interval. Conditional logistic regression was adjusted for age at COVID-19 diagnosis, hypertension, interstitial pneumonia and cancer.

We performed analyses restricted to patients with CIA; 102 patients with CIA were compared to 408 propensity score-matched controls (Table 5), but we found no increased probability of severe COVID-19 in CIA patients as compared with non-AIRD matched patients [aOR = 1.11 (0.68–1.81)].

**TABLE 5:** Odds of severe COVID-19: Comparison between patients with or without chronic inflammatory arthritis–case control study within EDS database.

	CIA patients (*N* = 102)	Controls (*N* = 408)	ASD	aOR	95% CI	*p*
**Severe COVID-19**	38 (37.3%)	141 (34.6%)		1.111	**[0.68–1.81]**	**0.67**
**Death**	18 (17.6%)	83 (20.3%)		1.001	**[0.55–1.81]**	**1**
**ICU admission**	20 (19.6%)	58 (14.2%)		1.2153	**[0.65–2.26]**	**0.54**
**Patient characteristics**						
Age	66.4 (16.1)	70.7 (17.3)	0.2573			
Sex: male	36 (35.3%)	138 (33.8%)	0.0309			
**Comorbidities**						
Interstitial lung disease	12 (11.8%)	31 (7.6%)	0.1413			
COPD	9 (8.8%)	39 (9.6%)	0.0255			
Cardiovascular diseases	40 (39.2%)	171 (41.9%)	0.0549			
Stroke	12 (11.8%)	57 (14.0%)	0.0659			
Diabetes	21 (20.6%)	89 (21.8%)	0.0300			
Obesity	26 (25.5%)	94 (23.0%)	0.0572			
Hypertension	62 (60.8%)	269 (65.9%)	0.1070			
Smoking	14 (13.7%)	55 (13.5%)	0.0071			
Cancer	20 (19.6%)	106 (26.0%)	0.1523			

ASD, absolute standardized difference; CIA, chronic inflammatory arthritis; COPD, chronic obstructive pulmonary disease; aOR, adjusted odds ratio; 95% CI, 95% confidence interval. Bold values represent the significantive value.

## Discussion

This observational, multicentric, French cohort study examined the frequency and predictors of severe COVID-19 in adults with AIRD. It included ambulatory and hospitalized patients covering the entire spectrum of the disease in this population. We confirmed that in AIRD patients, age, male sex, ILD, hypertension, obesity, and corticosteroids or rituximab use were associated with COVID-19 severity. Also, we identified age, ILD, hypertension, and corticosteroids or rituximab use as predictors of death in patients hospitalized with COVID-19. Finally, in our propensity score-matched case–control study, as compared with the general population, AIRD patients had higher risk of severe COVID-19 independent of their comorbidities. However, analyses restricted to CIA patients showed that these patients were not at increased risk of severe COVID-19. To our knowledge, few studies have compared patients with AIRD and matched controls (16–19), especially with a large number of patients.

Our study confirmed that older age, male sex, hypertension, obesity and ILD were associated with COVID-19 severity, in line with previous cohorts of COVID-19 in AIRD patients (11, 20–22) and in the general population (23–31). In the same way, predictors associated with death at 90 days (older age, hypertension or ILD) were concordant with the existing literature for the general population (32, 33), and with recent reports of the Global Rheumatology Alliance (10), and Japanese experience in 1,915 AIRD patients (34).

The association between ILD and severity of COVID-19 pneumonia might be explained by patients with previous injured pulmonary parenchyma being more susceptible to progression to severe COVID pneumonia because of impaired baseline pulmonary dysfunction and limited reserves. Moreover, COVID-19 could lead to an exacerbation of the ILD (27, 35–38). In the literature, the risk of death range from 30 to 60% for COVID-19 patients with pre-existing ILD, with ORs from 3.2 to 5.5 (32, 39, 40).

In our study, sarcoidosis, vasculitis and auto-inflammatory diseases were associated with severe COVID-19. These results confirmed those from the French RMD COVID-19 cohort study (5), that included some of our patients. However, here we had a much larger number of cases to confirm these findings: sarcoidosis (64 vs. 15), vasculitis (167 vs. 65) and auto-inflammatory disease (34 vs. 27). In sarcoidosis, two studies suggested that this association could be explained by ILD due to the disease, (41), with a specific increased risk of hospitalization in patients with stage III sarcoidosis (5, 42). Other underpowered cohorts did not identify sarcoidosis as a risk factor of severe COVID-19 (42, 43). Regarding vasculitis, in the absence of control populations, only few studies analyzed the risk of COVID severity associated with the vasculitis itself. The French RMD COVID-19 (5) and the Global Rheumatology Alliance (11) reported increased risk of severe or moderate COVID-19 requiring hospitalization. Nevertheless, these studies relied on a very low number of patients: 65 and 44, respectively. A few other cohorts have shown increased risk of death for patients with vasculitis, but the data are scarce and lack power (44). Additionally, in another cohort, the risk of severe COVID-19 was not higher in auto-inflammatory patients than in patients with other rheumatic conditions (45) or in the general population (46).

Use of corticosteroids and rituximab was associated with increased probability of severe COVID-19 or death. Overall, immunosuppressed patients are at increased risk of COVID-19 and severe COVID-19, even fully vaccinated individuals (47). However, this risk seems restricted to only some treatments, such as rituximab (10, 17, 34, 48–53), corticosteroids (5, 11, 19, 20, 22, 33, 54, 55), mycophenolate acid (5, 10), or cyclophosphamide (10), whereas other treatments, such as anti-tumor necrosis factor α, seem to be safe (11, 56). The negative impact of oral corticosteroids whatever the indication is well described (5, 11, 19, 20, 22, 33, 54, 55). In AIRD patients, the odds of hospitalization for COVID-19 were increased for those taking≥10 mg prednisone equivalent [aOR = 2.05 (1.06–3.96)] (11). This dose of corticosteroids was also found as an independent factor for COVID-19–related death (10). In CIA, this risk was not constantly observed (10, 57). Our result concerning the odds of corticosteroids with COVID-19 severity in our CIA patients, higher than in all AIRD patients, is puzzling. The difference could be explained by the other diseases included in our CIA category such as PsA and SA compared to RA patients alone. In contrast, the efficacy of high dose glucocorticoids to treat COVID-19 was demonstrated in the RECOVERY trial (58). The cause of this dichotomy may be related to the timing of use in relation to COVID-19 diagnosis (59).

The description of the high prevalence of COVID-19 and poorest outcome with rituximab is well described (17, 48–53). With COVID-19, the use of rituximab could lead to a persisting viremia without low viral clearance (60–64). In addition, even if our study was performed before the era of COVID-19 vaccination, anti-CD20 are well known to be associated with poor vaccinal response (65–67), so this treatment is still potentially risky in the era of vaccination. The impact of rituximab on COVID-19 prognosis might also explain, partly, the increased risk of severe COVID-19 in patients with vasculitis, who often receive rituximab. Nevertheless, even on multivariate analyses adjusted for underlying disease and in the analysis restricted to CIA, rituximab was still associated with increased probability of severity, with an even higher OR [5.20 (1.83;14.01)].

### Role of AIRDs in COVID-19 severity

One of the strengths of our study was the propensity score-matched case–control study that allowed for controlling for confounding factors such as age, sex, and comorbidities and analyzing specifically the risk of COVID-19 severity associated with the underlying AIRD. Indeed, patients without AIRD were younger, often male and had fewer comorbidities than those with AIRDs, so a crude comparison would have led to highly biased results. Overall, patients with AIRD were at increased probability of severe COVID-19 and borderline increased risk of death.

In a previous study, D’Silva et al. found that patients with AIRD had the same odds of hospitalization and mortality but 3-fold higher odds of ICU admission as compared with those without rheumatic disease [aOR = 3.11 (1.07–9.05)] (17). However, this study relied on a small number of patients (*n* = 52), and AIRD patients were analyzed together, without an analysis restricted to CIA patients. In another study, Pablo et al. did not find any overall increased risk of severe COVID-19 in 228 AIRD patients analyzed altogether [risk ratio = 1.13 (0.84–1.49)] but found an increased risk of severe COVID-19 in patients with other AIRDs (connective tissue diseases, vasculitis, etc.) as compared with CIA patients (16). However, in these cohorts, non-AIRD controls were matched on only age and sex but not comorbidities, which are much more frequent in AIRD patients, as demonstrated by our results. Regarding specifically patients with RA of SpA, as in our study, in the Mena-Vasquez et al. study, 78 patients with CIA did not have increased risk of severe COVID-19 as compared with age-, sex-, hypertension- and diabetes-matched controls (18). By contrast, one study found that patients with RA were at higher risk of severe COVID-19 (hospitalization or death) compared with non-RA controls matched on sex and age (57). But, again controls were not matched on comorbidities and it is possible that, during the first months of COVID-19 breakout, physicians were more prone to hospitalize RA patients on immunosuppressants, even though not having severe COVID-19, since they thought that they were at risk of severe forms. Although we had a smaller sample, our methodology was less subject to bias, and we did not find any increased risk of severe COVID-19 [aOR = 1.11 (0.68–1.81)] or death [aOR = 1 (0.55–1.8)] in CIA patients. Finally, none of these studies found increased risk for COVID-19-related death, but they could lack power (68).

### Limitations and strengths

Our study has some limitations. First, because of its retrospective nature, data collection might be subject to potential biases. However, this might have affected all groups of patients and both cases and controls in the same way. In addition, we had access to medical files of all patients, which allowed us to confirm all diagnoses of underlying AIRDs, comorbidities, treatments, and outcome in the first part of the study and to analyze the accuracy of our automated algorithms we developed in the second part. The use of an exhaustive database with an automatic search and a review by an investigator limits the risk of missing data and ensure its accuracy. Secondly, the utilization of two different sources can lead to a reporting bias. However, in both cases information was obtained from clinicians in charge of the patients, which is likely to minimize this bias. Finally, the data were collected before the development of COVID-19 vaccines and the emergence of virus variants and may not reflect the current situation, but studies of patients included after the vaccination period found the same factors as our study (34). Nevertheless, our study has several strengths. We were able to recruit a large patient population assessed within multiple centers in a single country. Moreover, we were able to collect patients with ambulatory and hospitalized forms of COVID-19, covering the entire clinical spectrum of the disease. Ambulatory patients are rarely considered in COVID-19 studies. Second, the use of the EDS-COVID database allowed us to run a propensity score-matched case–control study, including cases and controls from the same population and controlling for age and sex but also all comorbidities known to be associated with COVID-19 severity, which has never been done properly before.

## Conclusion

In this multicenter study performed before the era of COVID-19 vaccines, we confirmed that AIRD patients receiving rituximab or corticosteroids were at increased risk of severe COVID-19, as were those with vasculitis, auto-inflammatory disease, and sarcoidosis. Also, as compared with non-AIRD controls from the same cohort of hospitalized patients, AIRD patients had an overall increased odds of severe COVID-19 and a borderline increased odd of death. However, a reassuring point is that these increased odds were not observed in an analysis restricted to patients with RA or SpA.

## Data availability statement

The original contributions presented in this study are included in the article/Supplementary material, further inquiries can be directed to the corresponding author.

## Ethics statement

This present study was approved by the institutional review board (APHP Scientific and Ethical Committee, authorization no. CSE 20-60_CovAID) from the Scientific and Ethical Committee of the AP-HP and by the CNIL. Written informed consent for participation was not required for this study in accordance with the national legislation and the institutional requirements.

## Author contributions

KC collected the data, analyzed them, and wrote the manuscript. RS, XM, and EH designed the study. MG and TJ performed the statistics and help for the data collection. JA, R-MF, SG-L, SE, EP, TP, AS, HM, FD, PC, MD, AM, JS, BF, DR, EE, NC-C, and CR collected data from the patients. All authors reviewed and corrected the manuscript.
