# Supplementary material for: CovAID: Identification of factors associated with severe COVID-19 in patients with inflammatory rheumatism or autoimmune diseases
Source: Front Med (Lausanne). 2023 Mar 22;10:1152587. doi: 10.3389/fmed.2023.1152587 (PMC10075312; doi:10.3389/fmed.2023.1152587)
Supplement: Supplementary file 1 [file Data_Sheet_1.docx]

**Supplementary material**

[Supplemental 1: Material and methods 2](#_Toc121508952)

[Data source 2](#_Toc121508953)

[Table S1 – Autoimmune/inflammatory rheumatic disease (AIRD) of interest and their International Classification of Disease, 10th revision (ICD-10) code 3](#_Toc121508954)

[Table S2 – WHO criteria for severe COVID-19 4](#_Toc121508955)

[Table S3 – Collected comorbidities and their ICD-10 code 5](#_Toc121508956)

[Supplemental material 2: Performance of the PyMedExt Python library for comorbidity extraction 6](#_Toc121508957)

[Figure S1 – Specificity, sensitivity, precision and F1-score computed on the case cohort for each comorbidity of interest 7](#_Toc121508958)

[Table S4 – Specificity, sensitivity, precision and F1-score computed on the case cohort for each comorbidity of interest 7](#_Toc121508959)

[Supplemental 3: Identification of complications related to COVID-19 8](#_Toc121508960)

[Table S5: Factors associated with odds of severe COVID-19 in patients with chronic inflammatory arthritis: pooled analysis of EDS-COVID and French RMD cohorts 9](#_Toc121508961)

[Table S6: Factors associated with odds of COVID-19-related death in patients with chronic inflammatory arthritis: pooled analysis of EDS-COVID and the French RMD cohort 11](#_Toc121508962)

# Supplemental 1: Material and methods

## Data source

***EDS-COVID Database***

The Assistance Publique-Hopitaux de Paris (AP-HP) Health Data Warehouse (“Entrepôt de Données de Santé (EDS),” is an analytics platform collecting clinical data repository (CDR) from patients hospitalized or followed in the AP-HP hospitals since 2014. APHP is the largest hospital entity in Europe with 39 hospitals (22,474 beds), 1.5 million hospitalizations per year (10% of all hospitalizations in France) and 8.3 million patients per year. Since 2014, clinical data from patients were captured in clinical databases. The CDR has received the authorization of the French Data Protection Authority (Commission Nationale de l’Informatique et des Libertés, CNIL, no. 1980120).

From the beginning of COVID-19 epidemic, the EDS-COVID database emerged to collect information on patients admitted to APHP hospitals for COVID-19. Specifically, the condition for a patient to be included in the warehouse “EDS-COVID” was one of the following:

- the patient has a visit with a COVID-19-related associated International Classification of Disease, 10th revision (ICD-10) code (U07.1)
- the patient took a RT-PCR or a serology test
- COVID-19 was mentioned in the admission note of the patient

***The French RMD COVID-19 cohort***

The French RMD COVID-19 cohort is a French national, observational, multi-center cohort including patients from all ages with a confirmed inflammatory rheumatic and musculoskeletal disease. All patients including in this cohort were informed about the study, and patient consent was obtained for the use of medical data.

# Table S1 – Autoimmune/inflammatory rheumatic disease (AIRD) of interest and their International Classification of Disease, 10th revision (ICD-10) code

| **Group of diseases** | **Disease** | **ICD-10 code** |
| --- | --- | --- |
| **Chronic inflammatory rheumatism** | Rheumatoid arthritis | M05, M050, M0500-M0509, M051, M0510-M0519, M052, M0520-0529, M053, M0530-M0539, M058, M0580-M0589, M059, M0590-M0599, M06, M060-M0609M062, M0620-M0629, M063, M0630-M0639, M064, M0640-M0649, M068, M0680-MM0689, M069, M0690-M0699, |
|  | Axial and peripheral spondyloarthritis | M45, M45+0-M45+9 |
|  | Psoriatic arthritis | M07.0-M07.3*, M09.0*  M07, M0700, M0704, M0707, M0709, M072, M0720, M0728, M073, M0730-M0739, M074, M0740-M0749, M075, M0750-M0759, M076, M0760-M0769 |
| **Auto-inflammatory diseases** | Familial mediterranean fever | E85.0 |
|  | Still’s disease | M061, M0610-M0619 |
| **Sarcoidosis** | Sarcoidosis | D86, D860-D863, D868, D869 |
| **Vasculitis** | Vasculitis associated with cytoplasmic antineutrophil antibodies | M301, M313, M317 |
|  | Anti-MBG vasculitis | M310 |
|  | Giant cell arteritis | M315, M316 |
|  | Polymyalgia rheumatica | M353 |
|  | Takayasu's arteritis | M314 |
|  | Behcet’s disease | M352 |
|  | Other vasculitis | M30, M308, M31, M318, M319 |
| **Connectivitis** | Systemic lupus erythematosus | L93, L930-L932  M32, M320-M329 |
|  | Sjögren’s syndrome | M350 |
|  | Inflammatory myopathy (including dermatomyositis, polymyositis) | M33, M330—M332, M339, M360 |
|  | Systemic sclerosis | M34, M340—M342, M348, M349 |
|  | Mixed connective tissue disease | M35.1 |
|  | Undifferentiated connective tissue disease | M35.1 |

# Table S2 – WHO criteria for severe COVID-19

| **Patient state** | **Description** | **Score** |
| --- | --- | --- |
| **Uninfected** | Uninfected; no viral RNA detected | 0 |
| **Ambulatory mild disease** | Asymptomatic; viral RNA detected | 1 |
|  | Symptomatic; independent | 2 |
|  | Symptomatic; assistance needed | 3 |
| **Hospitalized; moderate disease** | Hospitalized; no oxygen therapy | 4 |
|  | Hospitalized; oxygen by mask or nasal prongs | 5 |
| **Hospitalized; severe diseases** | Hospitalized; oxygen by NIV of high flow | 6 |
|  | Intubation and mechanical ventilation, pO_2_/FiO_2_ ≥ 150 or SpO_2_/FiO_2_ ≥ 200 | 7 |
|  | Mechanical ventilation, pO_2_/FiO_2_ < 150 (SpO_2_/FiO_2_ < 200) or vasopressors | 8 |
|  | Mechanical ventilation, pO_2_/FiO_2_ < 150 and vasopressors, dialysis, or ECMO | 9 |
| **Dead** | Dead | 10 |

ECMO: extracorporeal membrane oxygenation; NIV: non-invasive ventilation

# Table S3 – Collected comorbidities and their ICD-10 code

| **Comorbidity** | **ICD-10 code** |
| --- | --- |
| Interstitial lung disease | J84 |
| Chronic obstructive pulmonary disease | J44 |
| Cardiovascular diseases | I21, I22, I23, I6, I50 |
| Stroke | I64 |
| Diabetes | E10, E11, E12, E13, E14 |
| Obesity | E66 |
| Hypertension | I10, I11 |
| Smoking | F17 |
| Cancer | C00 à C99, D0 à D49 |

# Supplemental material 2: Performance of the PyMedExt Python library for comorbidity extraction

Used metrics:

- Sensitivity 🡪 $\frac{TP}{TP+FN}$ Percentage of patients **with** a given comorbidity who were correctly identified as such
- Specificity 🡪 $\frac{TN}{TN+FP}$ Percentage of patients **without** a given comorbidity who were correctly identified as such
- Precision 🡪 $\frac{TP}{TP+FP}$ Percentage of patients **identified with** a given comorbidity who really had the comorbidity
- F1 Score 🡪 **Geometric mean** of precision and recall

TP = true positive, TN = True negative, FP = False positive and FN = False negative.

# Figure S1 – Specificity, sensitivity, precision and F1-score computed on the case cohort for each comorbidity of interest


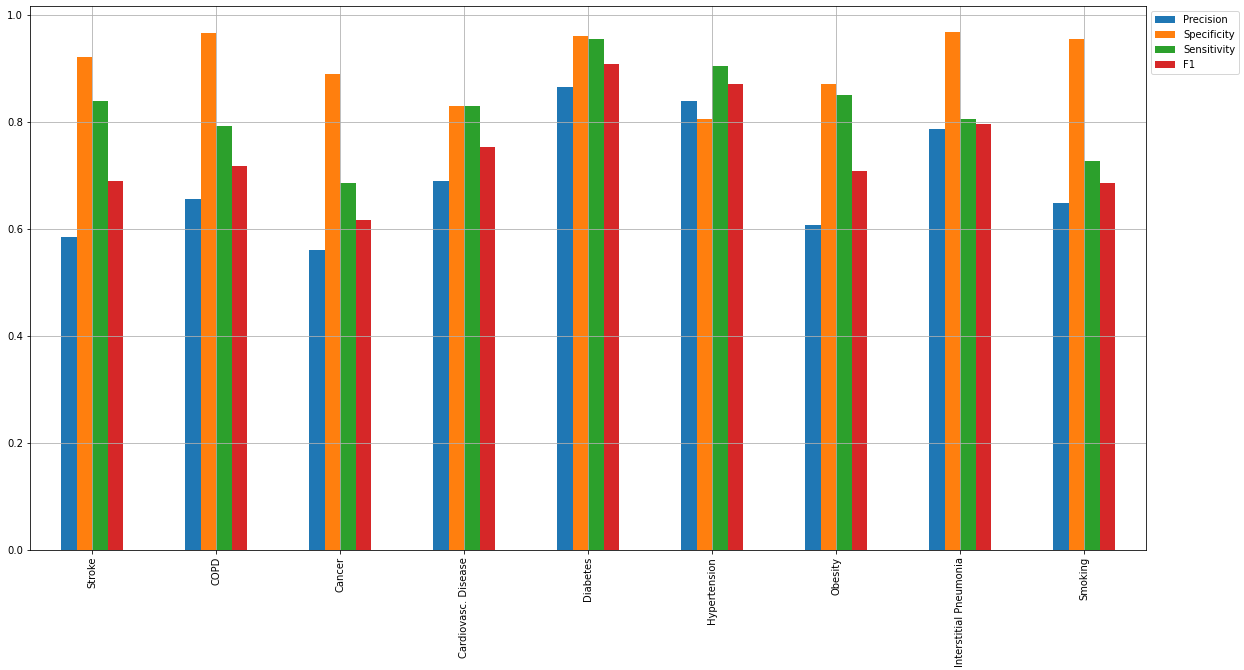


# Table S4 – Specificity, sensitivity, precision and F1-score computed on the case cohort for each comorbidity of interest

| **Comorbidity** | **Precision** | **F1** | **Sensitivity** | **Specificity** |
| --- | --- | --- | --- | --- |
| Stroke | 0.58 | 0.69 | 0.84 | 0.92 |
| COPD | 0.66 | 0.72 | 0.79 | 0.97 |
| Cancer | 0.56 | 0.62 | 0.69 | 0.89 |
| Cardiovascular disease | 0.69 | 0.75 | 0.83 | 0.83 |
| Diabetes | 0.86 | 0.91 | 0.96 | 0.96 |
| Hypertension | 0.84 | 0.87 | 0.9 | 0.81 |
| Obesity | 0.61 | 0.71 | 0.85 | 0.87 |
| Interstitial lung disease | 0.79 | 0.8 | 0.8 | 0.97 |
| Smoking | 0.65 | 0.69 | 0.73 | 0.95 |

# Supplemental 3: Identification of complications related to COVID-19

The date of COVID-19 diagnosis was used as the COVID+ date $T_{+}$. To compute the various patient variables of interest, we first tagged each hospital stay as COVID-related or not. This tagging involved comparing each stay starting and ending date ($t_{start}$and $t_{end}$ ) to $T_{+}$**.** We considered a stay as COVID-related with $t_{start}\in$ or and $t_{end}\geq T_{+-1}$, this second rule aiming specifically at targeting hospital-acquired COVID. Non-COVID-related stays were removed at this stage.

From these stays, we extracted stays that occurred in ICUs. Because multiple ICU stays could occur for a single patient, various variables were stored regarding those stays, namely:

- The number of ICU stays
- The start date of the first ICU stay
- The end date of the last ICU stay
- The length of the first ICU stay
- The time between the end of the last ICU stay and the start of the first ICU stay
- The total length - by summing - of all ICU stays

| Table S5: Factors associated with odds of severe COVID-19 in patients with chronic inflammatory arthritis: pooled analysis of EDS-COVID and French RMD cohorts  \|  \| **Overall** \| **Patients with mild or moderate disease** \| **Patients with severe disease** \| **OR (95% CI)** \| **p-value** \|  \| **aOR 95%CI** \| **p-value** \| \| --- \| --- \| --- \| --- \| --- \| --- \| --- \| --- \| --- \| \|  \| **N=690** \| **N=618** \| **N=72** \|  \|  \|  \|  \|  \| \| **Patient characteristics** \|  \|  \|  \|  \|  \|  \|  \|  \| \| Age \| 56.1 (15.9) \| 54.5 (15.4) \| 69.7 (12.8) \| 1.07 [1.05-1.09] \| <0.001 \|  \| **1.05[1.03-1.07]** \| **<0.001** \| \| Sex: male \| 456 (66.1%) \| 416 (67.3%) \| 40 (55.6%) \| 1.65 [1.00-2.69] \| 0.0478 \|  \| **2.19[1.25-3.87]** \| **0.0064** \| \| **Comorbidities** \|  \|  \|  \|  \|  \|  \|  \|  \| \| Interstitial lung disease \| 14 (2.03%) \| 8 (1.29%) \| 6 (8.33%) \| 7.02 [2.35-20.08] \| <0.001 \|  \| **4.26[1.22-14.39]** \| **0.0243** \| \| COPD \| 25 (3.62%) \| 20 (3.24%) \| 5 (6.94%) \| 2.38 [0.81-5.92] \| 0.1067 \|  \|  \|  \| \| Asthma \| 52 (7.54%) \| 47 (7.61%) \| 5 (6.94%) \| 0.98 [0.35-2.27] \| 0.9661 \|  \|  \|  \| \| Coronary heart diseases \| 68 (9.86%) \| 52 (8.41%) \| 16 (22.2%) \| 3.15 [1.66-5.75] \| <0.001 \|  \|  \|  \| \| Stroke \| 22 (3.19%) \| 16 (2.59%) \| 6 (8.33%) \| 3.57 [1.29-8.79] \| 0.0161 \|  \|  \|  \| \| Diabetes \| 69 (10.0%) \| 51 (8.25%) \| 18 (25.0%) \| 3.74 [2.02-6.73] \| <0.001 \|  \|  \|  \| \| Obesity \| 100 (17.7%) \| 87 (17.0%) \| 13 (25.5%) \| 1.71 [0.85-3.24] \| 0.1251 \|  \|  \|  \| \| Hypertension \| 187 (27.1%) \| 143 (23.1%) \| 44 (61.1%) \| 5.17 [3.14-8.66] \| <0.001 \|  \| **2.68[1.48-4.94]** \| **0.0011** \| \| Smoking \| 68 (9.86%) \| 64 (10.4%) \| 4 (5.56%) \| 0.56 [0.18-1.37] \| 0.2233 \|  \|  \|  \| \| Cancer \| 34 (4.93%) \| 27 (4.37%) \| 7 (9.72%) \| 2.46 [0.99-5.49] \| 0.0533 \|  \|  \|  \| \| No. of patients with at least 1 comorbidity \| 380 (59.5%) \| 316 (55.5%) \| 64 (91.4%) \| 7.95 [3.75-20.00] \| <0.001 \|  \|  \|  \| \| **Ongoing rheumatic diseases or AIRD treatments** \|  \|  \|  \|  \|  \|  \|  \|  \| \| Corticosteroids \| 156 (22.6%) \| 117 (18.9%) \| 39 (54.2%) \| 5.03 [3.05-8.36] \| <0.001 \|  \| **2.44[1.38-4.30]** \| **0.0022** \| \| Dose \| 5.00 [5.00;9.00] \| 5.00 [5.00;8.00] \| 5.00 [5.00;10.0] \| 1.04 [1.00-1.09] \| 0.0552 \|  \|  \|  \| \| NSAIDs \| 89 (12.9%) \| 87 (14.1%) \| 2 (2.78%) \| 0.22 [0.04-0.64] \| 0.0031 \|  \|  \|  \| \| Colchicine \| 3 (0.43%) \| 3 (0.49%) \| 0 (0.00%) \| NA \| NA \|  \|  \|  \| \| Hydroxychloroquine \| 18 (2.61%) \| 15 (2.43%) \| 3 (4.17%) \| 1.96 [0.50-5.79] \| 0.2983 \|  \|  \|  \| \| Methotrexate \| 303 (43.9%) \| 273 (44.2%) \| 30 (41.7%) \| 0.91 [0.55-1.48] \| 0.6951 \|  \|  \|  \| \| Leflunomide \| 41 (5.94%) \| 39 (6.31%) \| 2 (2.78%) \| 0.52 [0.11-1.59] \| 0.2819 \|  \|  \|  \| \| Salazopyrine \| 12 (1.74%) \| 10 (1.62%) \| 2 (2.78%) \| 2.06 [0.39-7.28] \| 0.3487 \|  \|  \|  \| \| Mycophenolate mofetil/mycophenolic acid \| 1 (0.14%) \| 1 (0.16%) \| 0 (0.00%) \| NA \| NA \|  \|  \|  \| \| Azathioprine \| 3 (0.43%) \| 2 (0.32%) \| 1 (1.39%) \| NA \| NA \|  \|  \|  \| \| IVIg \| 0 (0.00%) \| 0 (0.00%) \| 0 (0.00%) \| NA \| NA \|  \|  \|  \| \| Biologics \| 387 (56.1%) \| 364 (58.9%) \| 23 (31.9%) \| 0.33 [0.19-0.55] \| <0.001 \|  \|  \|  \| \| Anti-TNF alpha \| 266 (38.8%) \| 257 (41.8%) \| 9 (12.7%) \| 0.21 [0.10-0.41] \| <0.001 \|  \|  \|  \| \| Anti IL-6 \| 23 (3.35%) \| 21 (3.41%) \| 2 (2.82%) \| 0.99 [0.20-3.18] \| 0.9937 \|  \|  \|  \| \| Anti IL-1 \| 1 (0.15%) \| 1 (0.16%) \| 0 (0.00%) \| NA \| NA \|  \|  \|  \| \| Anti-IL17 \| 35 (5.10%) \| 33 (5.37%) \| 2 (2.82%) \| 0.63 [0.13-1.93] \| 0.4544 \|  \|  \|  \| \| Abatacept \| 20 (2.92%) \| 19 (3.09%) \| 1 (1.41%) \| 0.65 [0.07-2.61] \| 0.5948 \|  \|  \|  \| \| Rituximab \| 26 (3.79%) \| 18 (2.93%) \| 8 (11.3%) \| 4.32 [1.76-9.88] \| 0.0022 \|  \| **5.20[1.83-14.01]** \| **0.0026** \| \| JAK inhibitor \| 30 (4.35%) \| 24 (3.88%) \| 6 (8.33%) \| 2.37 [0.89-5.54] \| 0.0816 \|  \|  \|  \| \| Other \| 12 (1.75%) \| 12 (1.95%) \| 0 (0.00%) \| NA \| NA \|  \|  \|  \|   COPD: chronic obstructive pulmonary disease; IVIg: intravenous immunoglobulins; JAK: Janus kinase; NSAIDs: non-steroidal anti-inflammatory drugs; TNF: tumor necrosis factor; IL: interleukin |  |  |  | Univariate analyses |  |  | Multivariate analyse |
| --- | --- | --- | --- | --- | --- | --- | --- | --- | --- | --- | --- | --- | --- | --- | --- | --- | --- | --- | --- | --- | --- | --- | --- | --- | --- | --- | --- | --- | --- | --- | --- | --- | --- | --- | --- | --- | --- | --- | --- | --- | --- | --- | --- | --- | --- | --- | --- | --- | --- | --- | --- | --- | --- | --- | --- | --- | --- | --- | --- | --- | --- | --- | --- | --- | --- | --- | --- | --- | --- | --- | --- | --- | --- | --- | --- | --- | --- | --- | --- | --- | --- | --- | --- | --- | --- | --- | --- | --- | --- | --- | --- | --- | --- | --- | --- | --- | --- | --- | --- | --- | --- | --- | --- | --- | --- | --- | --- | --- | --- | --- | --- | --- | --- | --- | --- | --- | --- | --- | --- | --- | --- | --- | --- | --- | --- | --- | --- | --- | --- | --- | --- | --- | --- | --- | --- | --- | --- | --- | --- | --- | --- | --- | --- | --- | --- | --- | --- | --- | --- | --- | --- | --- | --- | --- | --- | --- | --- | --- | --- | --- | --- | --- | --- | --- | --- | --- | --- | --- | --- | --- | --- | --- | --- | --- | --- | --- | --- | --- | --- | --- | --- | --- | --- | --- | --- | --- | --- | --- | --- | --- | --- | --- | --- | --- | --- | --- | --- | --- | --- | --- | --- | --- | --- | --- | --- | --- | --- | --- | --- | --- | --- | --- | --- | --- | --- | --- | --- | --- | --- | --- | --- | --- | --- | --- | --- | --- | --- | --- | --- | --- | --- | --- | --- | --- | --- | --- | --- | --- | --- | --- | --- | --- | --- | --- | --- | --- | --- | --- | --- | --- | --- | --- | --- | --- | --- | --- | --- | --- | --- | --- | --- | --- | --- | --- | --- | --- | --- | --- | --- | --- | --- | --- | --- | --- | --- | --- | --- | --- | --- | --- | --- | --- | --- | --- | --- | --- | --- | --- | --- | --- | --- | --- | --- | --- | --- | --- | --- | --- | --- | --- | --- | --- | --- | --- | --- | --- | --- | --- | --- | --- | --- | --- | --- | --- | --- | --- | --- | --- | --- | --- | --- | --- | --- | --- | --- | --- | --- | --- | --- | --- | --- | --- | --- | --- | --- | --- | --- | --- | --- | --- | --- | --- | --- | --- | --- | --- | --- | --- | --- |

# Table S6: Factors associated with odds of COVID-19-related death in patients with chronic inflammatory arthritis: pooled analysis of EDS-COVID and the French RMD cohort

|  |  |  |  | **Univariate analyses** | | |  | **Multivariate analyse** | |
| --- | --- | --- | --- | --- | --- | --- | --- | --- | --- |
|  | [ALL] | Alive | Dead | OR (95% CI) | p-value | N |  | OR (95% CI) | p-value |
|  | N=253 | N=216 | N=37 |  |  |  |  |  |  |
| **Patient characteristics** |  |  |  |  |  |  |  |  |  |
| Age | 65.6 (15.5) | 63.8 (15.6) | 76.3 (9.59) | 1.07 [1.04-1.10] | <0.001 | 253 |  | **1.05 [1.03-1.07]** | **<0.001** |
| Sex: male | 91 (36.0%) | 79 (36.6%) | 12 (32.4%) | 0.85 [0.40-1.73] | 0.6559 | 253 |  | **2.19 [1.25-3.87]** | **0.0064** |
| **Comorbidities** |  |  |  |  |  |  |  |  |  |
| Interstitial lung disease | 10 (3.95%) | 5 (2.31%) | 5 (13.5%) | 6.51 [1.83;23.17] | 0.0048 | 253 |  | **4.26 [1.22-14.39]** | **0.0243** |
| COPD | 16 (6.32%) | 14 (6.48%) | 2 (5.41%) | 0.98 [0.19-3.40] | 0.9812 | 253 |  |  |  |
| Asthma | 23 (9.09%) | 20 (9.26%) | 3 (8.11%) | 0.97 [0.25-2.87] | 0.9632 | 253 |  |  |  |
| Coronary heart diseases | 55 (21.7%) | 44 (20.4%) | 11 (29.7%) | 1.68 [0.76-3.56] | 0.1941 | 253 |  |  |  |
| Stroke | 17 (6.72%) | 13 (6.02%) | 4 (10.8%) | 2.02 [0.59-5.89] | 0.244 | 253 |  |  |  |
| Diabetes | 51 (20.2%) | 40 (18.5%) | 11 (29.7%) | 1.89 [0.85-4.02] | 0.1159 | 253 |  |  |  |
| Obesity | 40 (22.3%) | 33 (21.3%) | 7 (29.2%) | 1.57 [0.58-3.89] | 0.3578 | 179 |  |  |  |
| Hypertension | 118 (46.6%) | 92 (42.6%) | 26 (70.3%) | 3.10 [1.51-6.74] | 0.0019 | 253 |  | **2.68[1.48-4.94]** | **0.0011** |
| Smoking | 18 (7.11%) | 16 (7.41%) | 2 (5.41%) | 0.86 [0.16-2.91] | 0.8217 | 253 |  |  |  |
| Cancer | 24 (9.49%) | 19 (8.80%) | 5 (13.5%) | 1.71 [0.57-4.49] | 0.3178 | 253 |  |  |  |
| No. of patients with at least 1 comorbidity | 194 (81.9%) | 159 (79.1%) | 35 (97.2%) | 6.31 [1.60-57.13] | 0.005 | 237 |  |  |  |
| **CIA treatments** |  |  |  |  |  |  |  |  |  |
| Corticosteroids | 95 (37.5%) | 71 (32.9%) | 24 (64.9%) | 3.69 [1.82-7.79] | <0.001 | 253 |  | **2.44 [1.38-4.30]** | **0.0022** |
| Dose | 5.00 [5.00;10.0] | 5.00 [5.00;9.00] | 6.00 [5.00;10.0] | NA | NA | 93 |  |  |  |
| NSAIDs | 16 (6.32%) | 16 (7.41%) | 0 (0.00%) | NA | NA | 253 |  |  |  |
| Colchicine | 2 (0.79%) | 2 (0.93%) | 0 (0.00%) | NA | NA | 253 |  |  |  |
| Hydroxychloroquine | 10 (3.95%) | 8 (3.70%) | 2 (5.41%) | 1.73 [0.32-6.59] | 0.4819 | 253 |  |  |  |
| Methotrexate | 119 (47.0%) | 105 (48.6%) | 14 (37.8%) | 0.65 [0.32-1.31] | 0.2309 | 253 |  |  |  |
| Leflunomide | 16 (6.32%) | 15 (6.94%) | 1 (2.70%) | 0.53 [0.06-2.26] | 0.4379 | 253 |  |  |  |
| Salazopyrine | 6 (2.37%) | 5 (2.31%) | 1 (2.70%) | 1.58 [0.16-8.24] | 0.6401 | 253 |  |  |  |
| Mycophenolate mofetil/mycophenolic acid | 1 (0.40%) | 1 (0.46%) | 0 (0.00%) | NA | NA | 253 |  |  |  |
| Azathioprine | 1 (0.40%) | 1 (0.46%) | 0 (0.00%) | NA | NA | 253 |  |  |  |
| IgIV | 0 (0.00%) | 0 (0.00%) | 0 (0.00%) | NA | NA | 253 |  |  |  |
| Biologics | 93 (36.8%) | 83 (38.4%) | 10 (27.0%) | 0.61 [0.27-1.27] | 0.193 | 253 |  |  |  |
| Anti-TNF alpha | 50 (20.0%) | 47 (22.0%) | 3 (8.33%) | 0.37 [0.10-1.03] | 0.0572 | 250 |  |  |  |
| Anti IL-6 | 4 (1.60%) | 3 (1.40%) | 1 (2.78%) | 2.55 [0.24-16.07] | 0.3779 | 250 |  |  |  |
| Anti IL-1 | 1 (0.40%) | 1 (0.47%) | 0 (0.00%) | NA | NA | 250 |  |  |  |
| Anti-IL17 | 8 (3.20%) | 8 (3.74%) | 0 (0.00%) | NA | NA | 250 |  |  |  |
| Abatacept | 8 (3.20%) | 7 (3.27%) | 1 (2.78%) | 1.17 [0.12-5.59] | 0.8663 | 250 |  |  |  |
| Rituximab | 15 (6.00%) | 11 (5.14%) | 4 (11.1%) | 2.45 [0.70-7.35] | 0.1506 | 250 |  | **5.20 [1.83-14.01]** | **0.0026** |
| JAK inhibitor | 10 (3.95%) | 7 (3.24%) | 3 (8.11%) | 2.83 [0.66-10.06] | 0.1474 | 253 |  |  |  |
| Other | 4 (1.60%) | 4 (1.87%) | 0 (0.00%) | NA | NA | 250 |  |  |  |

COPD: chronic obstructive pulmonary disease; IVIg: Intravenous immunoglobulins; JAK: Janus kinase; NSAIDs: non-steroidal anti-inflammatory drugs; TNF: tumor necrosis factor; IL: interleukin
