# Supplementary figures and images for: CovAID: Identification of factors associated with severe COVID-19 in patients with inflammatory rheumatism or autoimmune diseases
Source: Front Med (Lausanne). 2023 Mar 22;10:1152587. doi: 10.3389/fmed.2023.1152587 (PMC10075312; doi:10.3389/fmed.2023.1152587)

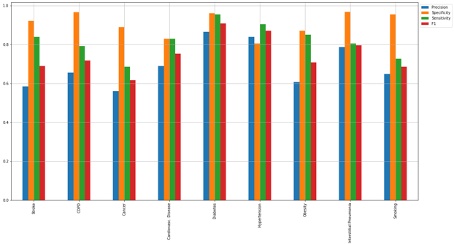

Supplement: Supplementary file 2 [file Image_1.jpeg]
